# Microbiota affects mitochondria and immune cell infiltrations via alternative polyadenylation during postnatal heart development

**DOI:** 10.3389/fcell.2023.1310409

**Published:** 2024-01-12

**Authors:** Xiang Liu, Yijia Shao, Linjiang Han, Yuanting Zhu, Jiazichao Tu, Jianrui Ma, Ruyue Zhang, Zhen Yang, Jimei Chen

**Affiliations:** ^1^ Department of Cardiac Surgery, Guangdong Cardiovascular Institute, Guangdong Provincial People’s Hospital (Guangdong Academy of Medical Sciences), Southern Medical University, Guangzhou, China; ^2^ Guangdong Provincial Key Laboratory of South China Structural Heart Disease, Guangzhou, China; ^3^ Department of Geriatrics, The First Affiliated Hospital, Sun Yat-sen University, Guangzhou, China; ^4^ Department of Emergency Medicine, The First Affiliated Hospital, Sun Yat-sen University, Guangzhou, China; ^5^ Department of Cardiology, The First Affiliated Hospital, Sun Yat-sen University, Guangzhou, China

**Keywords:** microbiota, heart development, alternative polyadenylation, mitochondrion, immune cell

## Abstract

There is a growing body of evidence supporting the significant impact of microbiota on heart development. Alternative polyadenylation (APA) is a crucial mechanism for gene expression regulation and has been implicated in postnatal heart development. Nonetheless, whether microbiota can influence postnatal heart development through the regulation of APA remains unclear. Therefore, we conducted APA sequencing on heart tissues collected from specific pathogen-free (SPF) mice and germ-free (GF) mice at three different developmental stages: within the first 24 h after birth (P1), 7-day-old SPF mice, and 7-day-old GF mice. This approach allowed us to obtain a comprehensive genome-wide profile of APA sites in the heart tissue samples. In this study, we made a significant observation that GF mice exhibited noticeably longer 3ʹ untranslated region (3ʹ UTR) lengths. Furthermore, we confirmed significant alterations in the 3ʹ UTR lengths of mitochondria-related genes, namely *Rala*, *Timm13*, and *Uqcc3*. Interestingly, the GF condition resulted in a marked decrease in mitochondrial cristae density and a reduction in the level of Tomm20 in postnatal hearts. Moreover, we discovered a connection between *Rala* and *Src*, which further implicated their association with other differentially expressed genes (DEGs). Notably, most of the DEGs were significantly downregulated in GF mice, with the exceptions being *Thbs1* and *Egr1*. Importantly, the GF condition demonstrated a correlation with a lower infiltration of immune cells, whereby the levels of resting NK cells, Th17 cells, immature dendritic cells, and plasma cells in GF mice were comparable to those observed in P1 mice. Furthermore, we established significant correlations between these immune cells and *Rala* as well as the related DEGs. Our findings clearly indicated that microbiota plays a vital role in postnatal heart development by affecting APA switching, mitochondria and immune cell infiltrations.

## Introduction

Microbiota has long been recognized as a pivotal factor in cardiovascular health and diseases (Tang et al., 2019). Studies have demonstrated that germ-free (GF) mice exhibit smaller hearts compared to their conventionally raised counterparts (Wostmann et al., 1982). Moreover, the colonization of the fetal gut during utero has been linked to cardiac growth (Guzzardi et al., 2019). Notably, a recent investigation highlighted the potential involvement of disturbances in maternal gut microbiota and plasma metabolites in the increased risk of congenital heart disease in offspring (Wang T. et al., 2021). These findings collectively imply that microbiota can influence fetal heart development. However, our current understanding of the impact of microbiota on postnatal cardiac development remains limited.

Alternative polyadenylation (APA) represents a prevalent post-transcriptional regulatory mechanism that influences gene expression by modulating the length of the 3ʹ untranslated region (3ʹ UTR) of each gene within the genome. Alterations in the length of the 3ʹ UTR impact the enclosed sequence and structural elements, thereby influencing mRNA stability, translation efficiency, and protein localization (Gruber and Zavolan, 2019). Previous studies have reported that elongation of 3ʹ UTRs can reduce gene expression, while shortening of 3ʹ UTRs can enhance mRNA stability and augment protein production (Mayr and Bartel, 2009; Chen et al., 2018). Noteworthy studies have indicated the pivotal role of alternative splicing in postnatal heart remodeling and development, specifically concerning genes like Camk2d, cTNT, and those involved in vesicular trafficking (Ladd et al., 2005; Xu et al., 2005; Giudice et al., 2014). However, it remains uncertain whether microbiota can influence postnatal heart development by regulating APA. Thus, further elucidation is required to understand the temporal dynamics of APA switching during postnatal heart development, both in the presence and absence of microbiota exposure.

This study was designed to investigate the potential impact of microbiota on APA switching in postnatal heart by using genome-wide sequencing. There is the hope that this research will increase the understanding of the effects of microbiota on postnatal heart development from the perspective of APA.

## Materials and methods

### Animal experiments

All experiments conform to the protocols approved by the Institutional Animal Care and Use Committee of the Guangdong Provincial People’s Hospital (No. KY-D-2021-396-01), in compliance with the National Research Council’s Guide for the Care and Use of Laboratory Animals. GF male C57BL/6J mice were born and bred at the GF Mouse Research Facility, the First Affiliated Hospital of Sun Yat-sen University. Specific pathogen free (SPF) male C57BL/6J mice were born and bred at the Laboratory Animal Centre of Sun Yat-sen University. SPF mice are free from a defined list of pathogens by routine testing and housed in clean conditions that minimize exposure to pathogens (Coughlan, 2021). Therefore, nonspecific microorganisms and parasites are allowed in SPF mice. By contrast, GF mice are free of all microorganisms (Parashar and Udayabanu, 2017). SPF mice have been widely adopted as the control group in GF mice-related studies (Llewellyn et al., 2018; Wang G. et al., 2021). Intraperitoneal anesthesia was administered by pentobarbital sodium (50 mg/kg), and then the heart tissues from P1, P7 and P7_GF mice were immediately dissected for subsequent studies.

### Detection of APA sites by sequencing

APA sequencing (*in vitro* transcription sequencing alternative polyadenylation sites, IVT-SAPAS) was performed to obtain the genome-wide profile of tandem 3′ UTRs as described previously (Fu et al., 2015; Fu et al., 2018; Liu et al., 2021). Total RNA was isolated using TRIzol reagent (Invitrogen, United States) and genome DNA was removed by Ambion Turbo DNA-free kit (Invitrogen, United States), then total RNA was randomly fragmented by heating. The first round of reverse transcription was performed using SuperScript III Kit (Thermo Fisher Scientific, MA, United States), and double-strand DNA was extracted with Agencourt RNAClean XP Kit (Beckman, CA, United States). The *in vitro* transcription RNA synthesis and purification were performed using RiboMAXTM Large Scale RNA Production Systems (Promega, WI, United States), and then the second round of reverse transcription and double-strand DNA purification were performed. After PCR amplification, fragments between 300 bp and 500 bp were selected with Agencourt AMPure XP beads (Beckman, CA, United States), and the library preparations were sequenced on an Illumina NovaSeq 6,000 platform.

### Sequencing data analysis

Sequencing data were analyzed with an in-house bioinformatics pipeline (Fu et al., 2015; Fu et al., 2018), as described in our previous study (Liu et al., 2021). Briefly, the raw reads were mapped to the mouse genome and then internal priming was filtered, and poly(A) sites were defined for each sample by clustering the unique mapped reads and then merged together across samples. Next, 3′ UTR length was standardized by designating the longest 3′ UTR as 1.0 and calculated the weighted mean length with multiple APA sites for each gene, and tandem 3′ UTR length difference between samples was detected by a test of linear trend alternative to independence. The Benjamin-Hochberg FDR was obtained, and FDR < 0.01 and | r | > 0.1 were set as the significant threshold. Additionally, the differentially expressed genes (DEGs) were calculated using limma package in R, and |log_2_ fold change| > 1 and *p* < 0.05 were set as the significant threshold.

### Functional annotation

For genes with switched 3′ UTRs, the functional annotations were performed by Metascape (Zhou et al., 2019), and the networks were visualized on Cytoscape in which the annotations were clustered by color and the size of each node depends on the *p*-value (Shannon et al., 2003). STRING database was used for analyzing the protein-protein interactions (PPI) (Szklarczyk et al., 2021), and the networks were visualized on Cytoscape in which the color depth of each node depends on the degree of interaction and the width of each edge depends on the edge betweenness (Shannon et al., 2003). For DEGs, Gene Ontology (GO) analyses were performed using clusterProfiler package in R. PPI were constructed in STRING database in which the disconnected nodes were hidden. The infiltrations of immune cell subsets were assessed by CIBERSORT algorithms with the reference of mouse signature (Chen et al., 2017). And the correlations between immune cells and key genes were analyzed via Hmisc package in R.

### Validation of the switching of tandem 3′ UTR lengths

Quantitative polymerase chain reaction (qPCR) was performed as previously described (Liu et al., 2021; Liu et al., 2022). Briefly, total RNA was isolated using TRIzol reagent (Invitrogen, United States) and concentration was measured using NanoDrop 2000 spectrophotometer (Thermo Fisher Scientific, United States). Then RNA was reversely transcribed into cDNA using Color Reverse Transcription Kit (EZBioscience, United States), and qPCR was performed on Bio-Rad CFX-96 (Bio-Rad, United States) with Color SYBR Green qPCR Master Mix (EZBioscience, United States). The 2^^-△CT^ value was calculated and the distal 2^^-△CT^ value was divided by the proximal 2^^-△CT^ value, and the results were normalized. The qPCR primers used in the study are listed in Supplementary Table S1.

### Transmission electron microscope

Heart tissues were rapidly immersed in 2.5% glutaraldehyde and trimmed to 1 mm^3^ in size, and fixed for 24 h in the refrigerator. After washing with 0.1 M phosphate buffer (PB), the samples were postfixed in 1% osmium tetroxide for 2 h, and washed with 0.1 M PB again. The samples were dehydrated with gradient ethanol (30%, 50%, 70%, 80%, 90% and 100%) and pure acetone, and embedded in embedding agent. Then ultrathin sections (80 nm) were obtained with an ultramicrotome and mounted on copper grids. The samples were stained with uranyl acetate and lead citrate, and observed by a transmission electron microscope.

### Immunofluorescence analysis

The procedure was performed as previously described (Liu et al., 2021). The heart tissues were fixed in 4% paraformaldehyde, and then dehydrated and embedded in paraffin. Samples underwent dewaxing and antigen retrieval. The slides were blocked in 10% goat serum for 30 min at room temperature and then incubated with primary antibodies of RALA (Affinity Biosciences, Jiangsu, China) and TOMM20 (Abcam, MA, United States) overnight. Slides were then incubated with Alexa Fluor^®^ 594 donkey anti-rabbit lgG (H+L). Wheat germ agglutinin (WGA, Sigma-Aldrich, United States) staining was used to outline the cardiomyocytes. Slides were then washed and stained with DAPI (Solarbio, Beijing, China). The positive signals were detected with a fluorescence microscope (Olympus, Tokyo, Japan).

### Statistical analysis

Data were presented as mean ± standard error of the mean (SEM). Statistical analysis was performed with IBM SPSS Statistics 20.0 (IBM, United States). The comparisons among groups were performed with one-way analysis of variance (ANOVA) followed by LSD test for pairwise comparisons. *p* < 0.05 was considered to be statistically significant.

## Results

### The profile of APA switching in postnatal hearts with or without microbiota exposure

**FIGURE 1:**
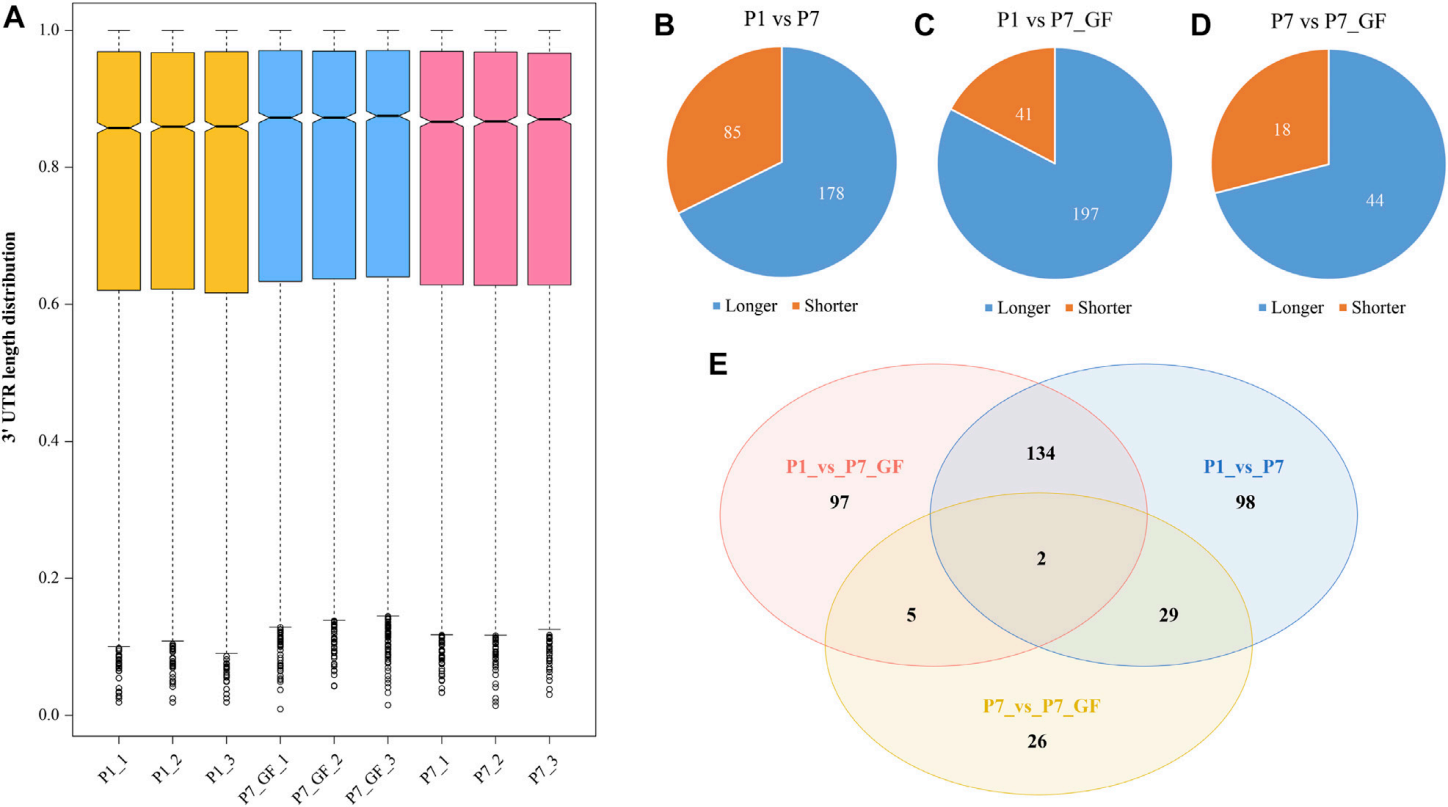
The profile of APA switching in postnatal hearts with or without microbiota exposure. **(A)** The normalized 3′ UTR length of each gene in each sample was shown. Overall, the 3′ UTRs in P7 mice became longer compared to those in P1 mice, and P7_GF mice have even longer 3′ UTRs compared to the conventional counterparts. **(B)** Compared to P1 mice, there were 263 (178 longer and 85 shorter) genes harboring switched 3′ UTRs in P7 mice. **(C)** Compared to P1 mice, there were 238 (197 longer and 41 shorter) genes harboring switched 3′ UTRs in P7_GF mice. **(D)** Compared to P7 mice, there were 62 (44 longer and 18 shorter) genes harboring switched 3′ UTRs in P7_GF mice. **(E)** There were two overlapping genes, and 98, 97 and 26 genes were found exclusively in P1_vs_P7, P1_vs_P7_GF and P7_vs_P7_GF, respectively.

GF and SPF male mice were adopted and the heart tissues isolated from SPF mice within the first 24 h after birth (P1), 7-day-old SPF mice (P7) and 7-day-old GF mice (P7_GF) were subjected to sequencing using the IVT-SAPAS method. A total of 181,765,525 raw reads with lengths of 75 bp were obtained, and 117,066,775 (64.4%) reads were uniquely mapped to the genome. By filtering the internal priming, 72,261,782 (39.8%) reads could be directly applied to the detection of polyadenylate [poly(A)] sites (Supplementary Table S2). Nearly three-quarters of the filtered reads (72.4%) mapped to the known poly(A) sites listed in the UCSC (56.6%) and polyA_DB database (15.8%) (Zhang et al., 2005) (Supplementary Figure S1A). As for the distribution of poly(A) signals, AATAAA and ATTAAA signals occurred most frequently, which accounted for 45.1% and 8.7%, respectively (Supplementary Figure S1B). Together, these results indicated that IVT-SAPAS can be served as an excellent approach to identify the poly(A) sites. The normalized 3′ UTR length of each gene in each sample was calculated and displayed in the boxplot (Figure 1A). Overall, the 3′ UTR lengths in P7 mice (0.772610 ± 0.235275) became longer compared to that in P1 mice (0.766775 ± 0.239368), and what’s more, the P7_GF mice (0.777086 ± 0.233986) harbored even longer 3′ UTR lengths compared to the conventional counterparts (*p* < 0.01). Compared to P1 mice, there were 263 (178 longer and 85 shorter) and 238 (197 longer and 41 shorter) genes harboring switched 3′ UTRs in P7 and P7_GF mice, respectively (Figures 1B, C). Compared to P7 mice, there were 62 (44 longer and 18 shorter) genes with switched 3′ UTRs in the GF counterparts (Figure 1D). And Venn diagram showed that there were two overlapping genes, and 98, 97 and 26 genes were found exclusively in P1_vs_P7, P1_vs_P7_GF and P7_vs_P7_GF, respectively (Figure 1E).

**FIGURE 2:**
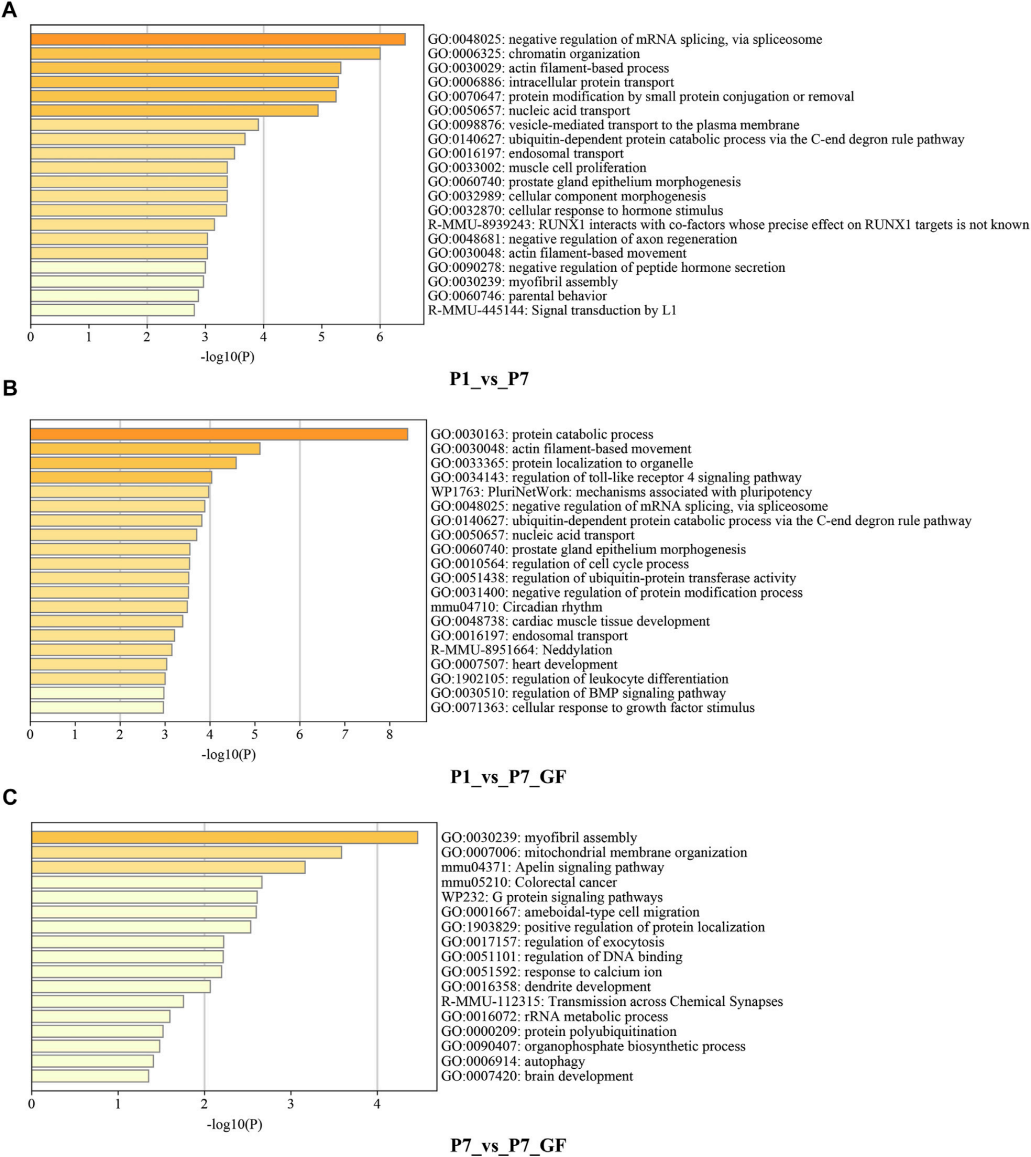
Functional annotations of the genes harboring switched 3′ UTRs. **(A)** Compared to P1 mice, genes with switched 3′ UTRs in P7 mice prominently enriched in the biological processes such as negative regulation of mRNA splicing via spliceosome, chromatin organization and actin filament-based process. **(B)** Compared to P1 mice, genes with switched 3′ UTRs in P7_GF mice were significantly related to protein catabolic process, actin filament-based movement and protein localization to organelle, etc. **(C)** Compared to P7 mice, genes with switched 3′ UTRs in P7_GF mice were notably enriched in myofibril assembly, mitochondrial membrane organization and Apelin signaling pathway, etc.

Functional annotations using Metascape were performed to investigate the biological significance of genes with switched 3′ UTRs. Compared to P1 mice, genes with switched 3′ UTRs in P7 mice exhibited significant enrichment in biological processes such as negative regulation of mRNA splicing via spliceosome, chromatin organization, and actin filament-based processes (Figure 2A). In contrast, genes with switched 3′ UTRs in P7_GF mice were found to be significantly associated with protein catabolic processes, actin filament-based movement, and protein localization to organelles (Figure 2B). Furthermore, comparison between P7 mice and their GF counterparts revealed that genes with switched 3′ UTRs were notably enriched in myofibril assembly, mitochondrial membrane organization, and the Apelin signaling pathway (Figure 2C). These findings highlight the distinct functional profiles of genes with switched 3′ UTRs, suggesting the importance of postnatal time and environment in modulating gene expression and function.

### Verification of the APA switching and mitochondria in postnatal hearts with or without microbiota exposure

**FIGURE 3:**
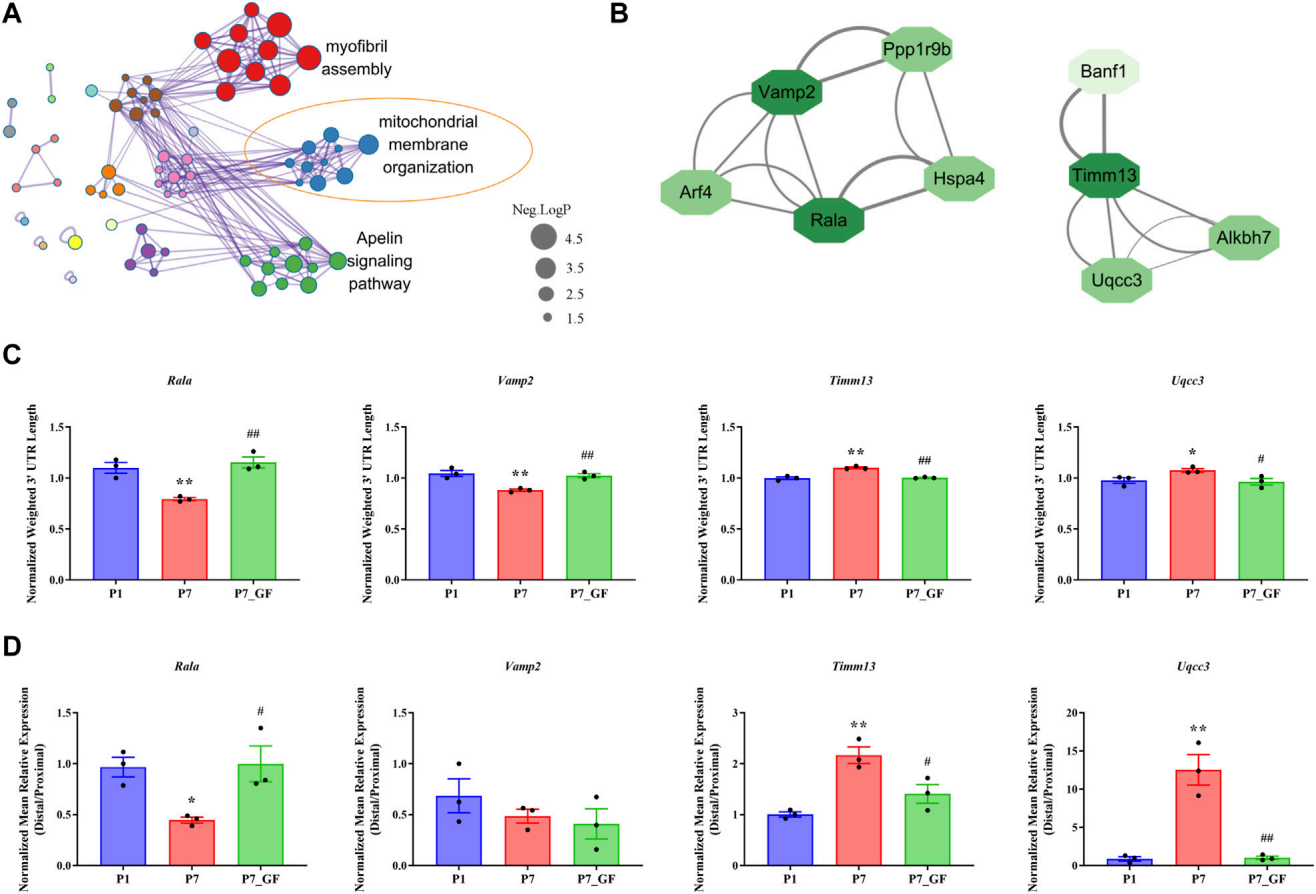
Verification of the APA switching genes related to mitochondria between P7 and P7_GF mice. **(A)** The functional annotations of genes with switched 3′ UTRs between P7 and P7_GF mice were visualized on Cytoscape. **(B)** The genes included in the module of mitochondrial membrane organization were selected for PPI analysis and *Rala*, *Vamp2*, *Timm13* and *Uqcc3* were identified as the key genes. **(C)** The 3′ UTR lengths of *Rala*, *Vamp2*, *Timm13* and *Uqcc3* were determined in sequencing. **(D)** The 3′ UTR lengths of *Rala*, *Timm13* and *Uqcc3* were further verified by qPCR. ^*^ *p* < 0.05, ^**^ *p* < 0.01 versus the P1 mice; ^#^ *p* < 0.05, ^##^ *p* < 0.01 versus the P7 mice. Data were expressed as mean ± SEM (*n* = 3).

**FIGURE 4:**
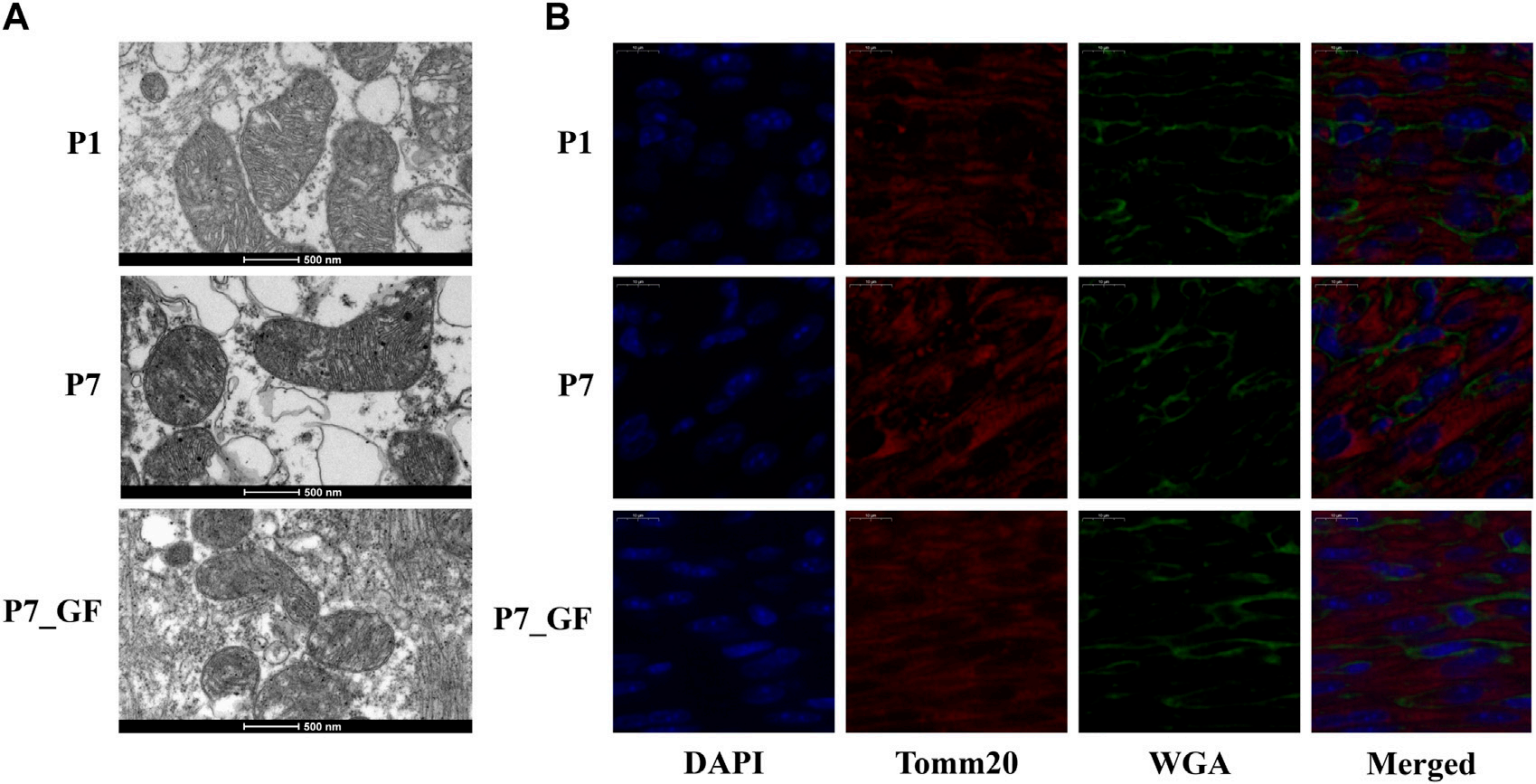
Verification of the changes in myocardial mitochondria with or without microbiota exposure. **(A)** Postnatal hearts of GF mice showed an obvious reduction in mitochondrial cristae density. **(B)** Representative immunofluorescence images showing expressions of Tomm20 in postnatal hearts. DAPI indicates nuclei and Wheat Germ Agglutinin (WGA) highlights the cell membrane.

To further understand how microbiota may influence postnatal heart development, the APA switching genes that enriched in the module of mitochondrial membrane organization (Figure 3A) were selected for PPI analysis. *Rala*, *Vamp2*, *Timm13* and *Uqcc3* were identified as the key genes (Figure 3B). And the variations in 3′ UTR lengths of *Rala*, *Timm13* and *Uqcc3*, as determined by sequencing (Figure 3C), were verified by qPCR (Figure 3D). The 3′ UTR length of *Rala* became shorter in P7 mice but completely restored in P7_GF mice. By contrast, the 3′ UTR lengths of *Timm13* and *Uqcc3* became longer in P7 mice but reversed in P7_GF mice. Furthermore, the myocardial mitochondria in conventional mice were found to be normal, but those in their GF counterparts showed an obvious reduction in mitochondrial cristae density (Figure 4A). Additionally, GF condition led to a lower level of Tomm20 in postnatal hearts (Figure 4B).

### The profile of differentially expressed genes in postnatal hearts with or without microbiota exposure

**FIGURE 5:**
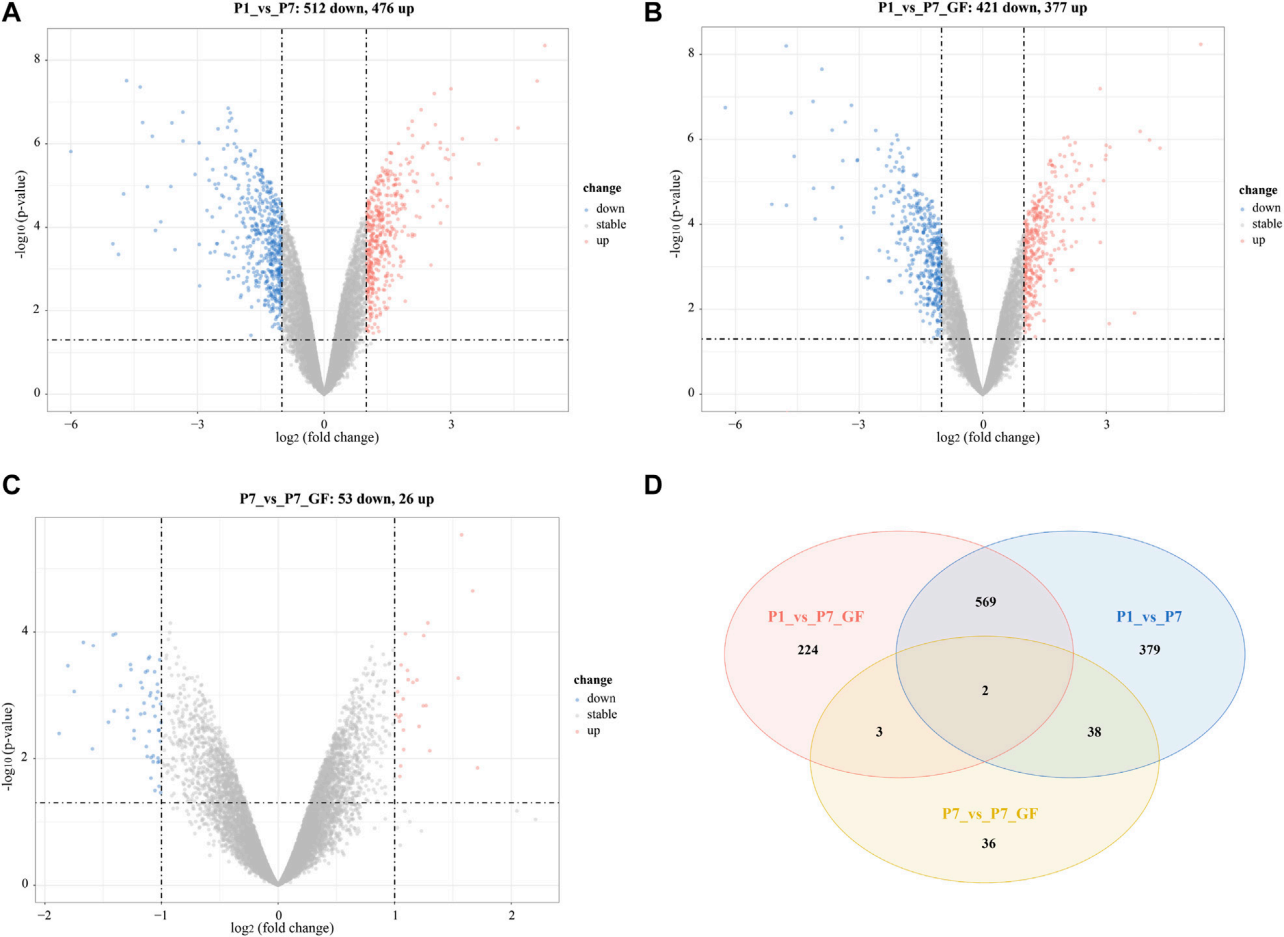
The profile of DEGs in postnatal hearts with or without microbiota exposure. **(A)** Compared to P1 mice, there were 988 (512 down and 476 up) DEGs in P7 mice. **(B)** Compared to P1 mice, there were 798 (421 down and 377 up) DEGs in P7_GF mice. **(C)** Compared to P7 mice, there were 79 (53 down and 26 up) DEGs in P7_GF mice. **(D)** There were two overlapping DEGs, and 379, 224 and 36 DEGs were found exclusively in P1_vs_P7, P1_vs_P7_GF and P7_vs_P7_GF, respectively. Upregulation and downregulation are presented as red and blue color in the Volcano plot, respectively.

In addition to identifying genes with switched poly(A) sites, we also obtained the profile of DEGs in postnatal hearts across different groups. A total of 11,777 genes were identified, with 5,492 genes (46.63%) harboring two or more tandem APA sites (Supplementary Figure S2). When comparing with P1 mice, we observed 988 DEGs in P7 mice (512 downregulated and 476 upregulated) (Figure 5A) and 798 DEGs in P7_GF mice (421 downregulated and 377 upregulated) (Figure 5B). And there were 53 downregulated and 26 upregulated DEGs in P7_GF mice when compared with their counterparts (Figure 5C). In addition, Venn diagram revealed that there were two overlapping DEGs, while 379, 224, and 36 DEGs were exclusively identified in the P1_vs_P7, P1_vs_P7_GF, and P7_vs_P7_GF comparisons, respectively (Figure 5D).

### Functional annotations and PPI analysis of the DEGs

**FIGURE 6:**
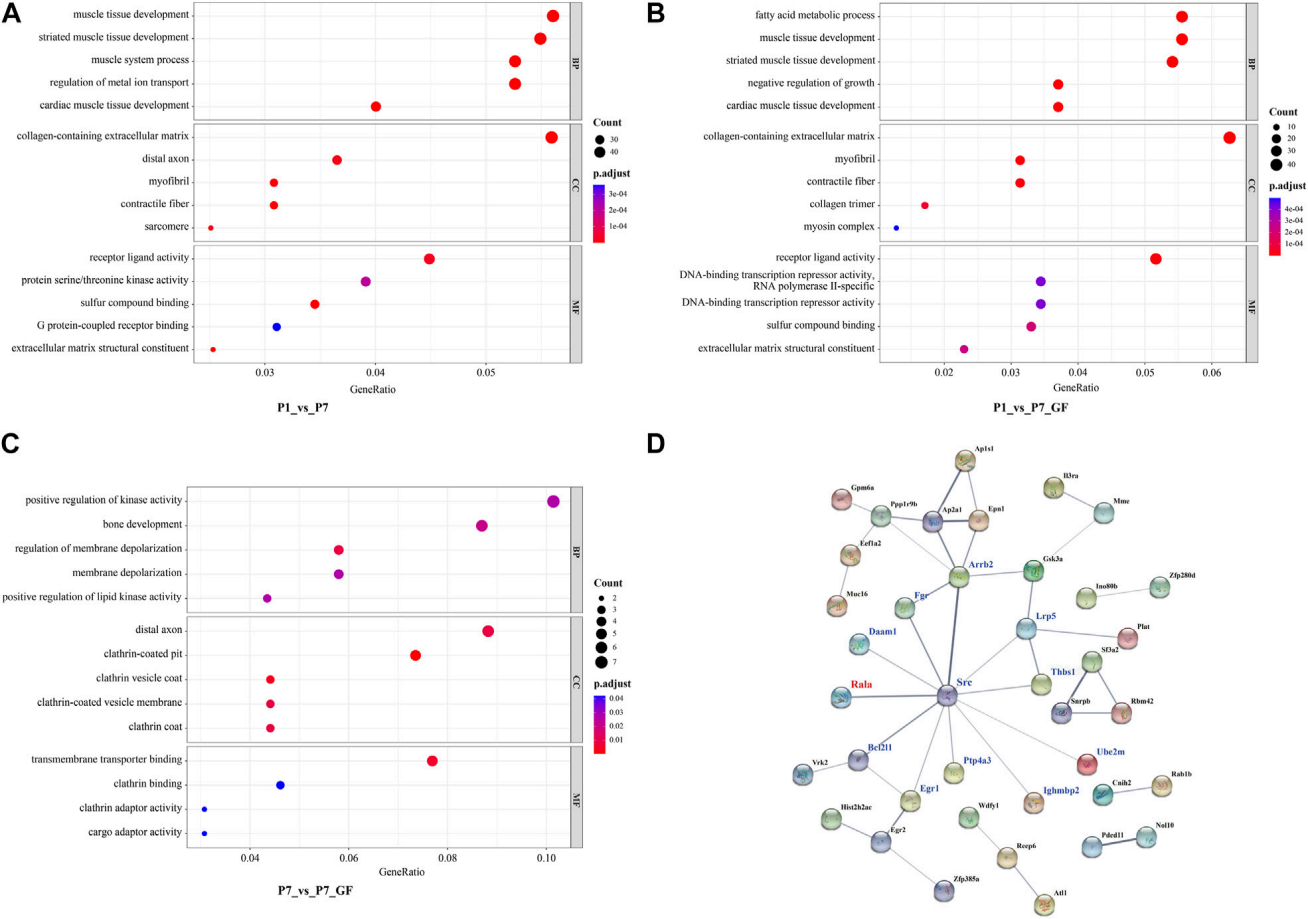
Functional annotations and PPI analysis of the DEGs. **(A, B)** P1_vs_P7 and P1_vs_P7_GF shared many common functional GO terms, such as muscle tissue development, collagen-containing extracellular matrix and receptor ligand activity. **(C)** DEGs in P7_vs_P7_GF were significantly enriched in positive regulation of kinase activity, distal axon and transmembrane transporter binding, etc. **(D)** PPI analysis of the DEGs in P7_vs_P7_GF and the verified APA switching genes (*Rala*, *Timm13* and *Uqcc3*) was conducted. It was found that only *Rala* can connect with another gene, namely *Src*, and then related to other DEGs. The top 5 GO terms are shown in the Bubble diagrams. The size of the dot represents gene count and the color illustrates adjusted *p*-value.

To gain further insights into the significance of these DEGs, GO enrichment analysis was conducted and the top 5 GO terms of biological processes, molecular functions and cellular components were shown. Since there were 571 overlapping genes between P1_vs_P7 and P1_vs_P7_GF, they shared many common functional GO terms, such as muscle tissue development, collagen-containing extracellular matrix and receptor ligand activity (Figures 6A, B). However, the functional profile of the P7_vs_P7_GF group differed significantly from the other two groups, such as positive regulation of kinase activity and transmembrane transporter binding (Figure 6C). Furthermore, PPI analysis was performed to explore the relationships between the DEGs in the P7_vs_P7_GF comparison and the validated APA switching genes (*Rala, Timm13, and Uqcc3*). The analysis revealed that *Rala* was the only gene that directly connected with another gene, namely *Src*, and subsequently associated with other DEGs, including *Daam1, Fgr, Arrb2, Lrp5, Thbs1, Ube2m, Ighmbp2, Ptp4a3, Egr1,* and *Bcl2l1* (Figure 6D).

### The expression levels of Rala and the related DEGs

**FIGURE 7:**
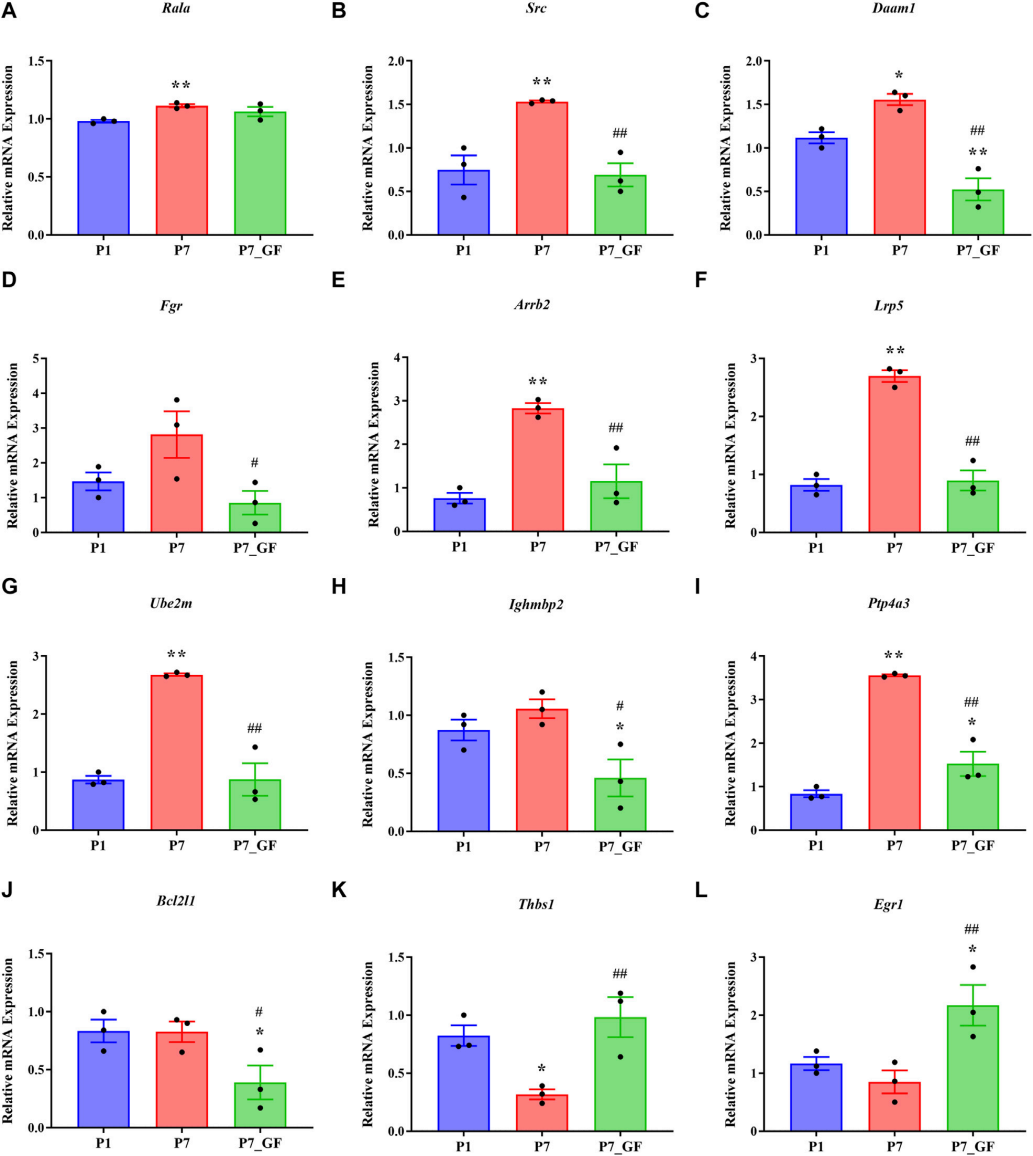
The expression levels of *Rala* and the related DEGs in sequencing. **(A)** The mRNA abundance of *Rala* remained unchanged between P7 and P7_GF mice. **(B–J)** Compared to P7 mice, the mRNA expressions of *Src*, *Daam1*, *Fgr*, *Arrb2*, *Lrp5*, *Ube2m*, *Ighmbp2*, *Ptp4a3* and *Bcl2l1* were notably downregulated. **(K, L)** Compared to P7 mice, the mRNA expressions of *Thbs1* and *Egr1* were dramatically upregulated. ^*^ *p* < 0.05, ^**^ *p* < 0.01 versus the P1 mice; ^#^ *p* < 0.05, ^##^ *p* < 0.01 versus the P7 mice. Data were expressed as mean ± SEM (*n* = 3).

**FIGURE 8:**
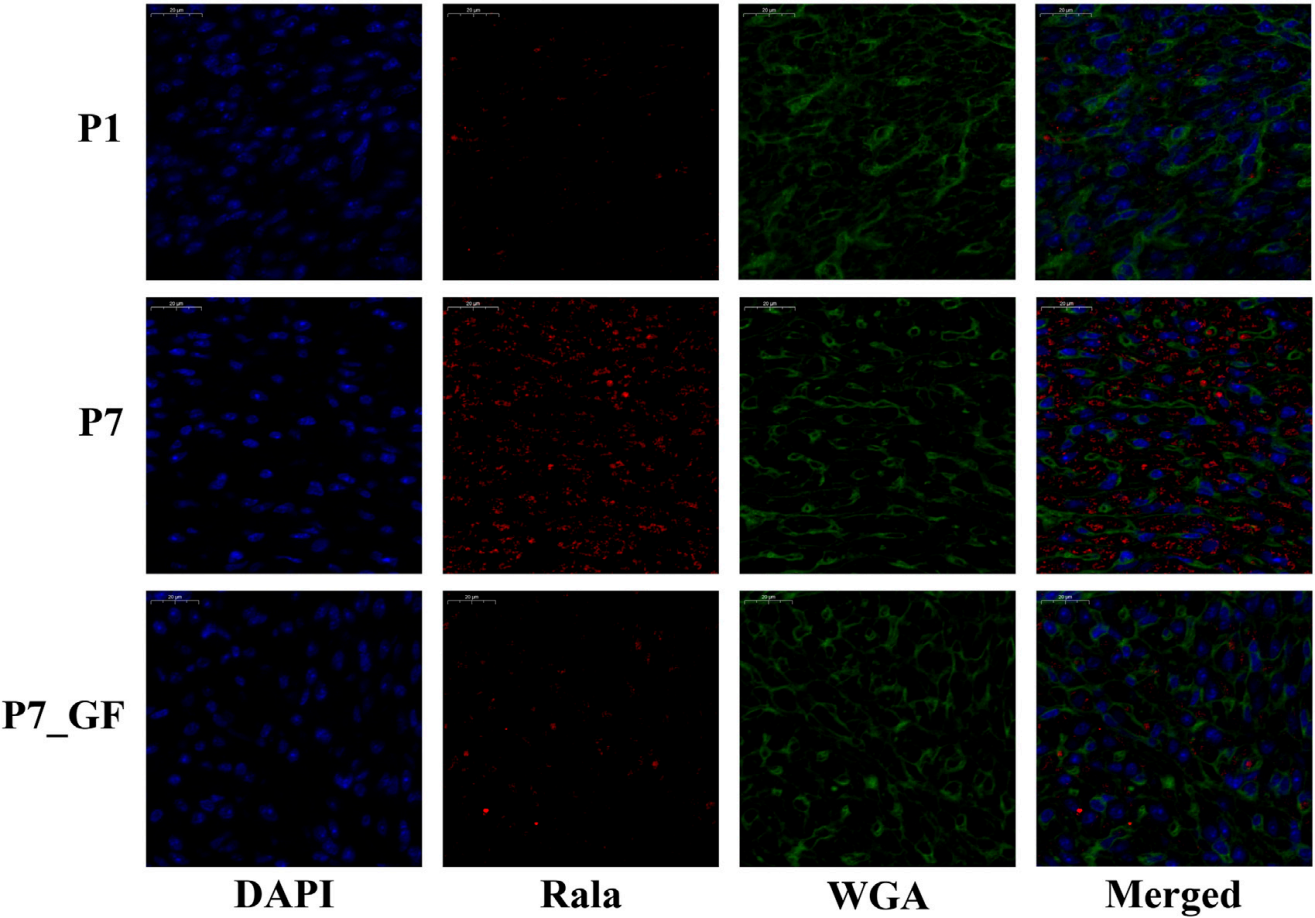
The protein levels of Rala were differentially expressed by microbiota exposure. Representative immunofluorescence images showing expressions of Rala in postnatal hearts. DAPI indicates nuclei and Wheat Germ Agglutinin (WGA) highlights the cell membrane.

Experimental evidence has demonstrated that the length of the 3′ UTR of *Rala* was significantly increased in P7_GF mice compared to the conventional counterparts, while the mRNA abundance remained unchanged between the two groups (Figure 7A). Conversely, notable changes were observed in the mRNA levels of other genes. Specifically, compared to P7 mice, the mRNA expressions of *Src, Daam1, Fgr, Arrb2, Lrp5, Ube2m, Ighmbp2, Ptp4a3, and Bcl2l1* were significantly downregulated (Figures 7B–J), whereas *Thbs1* and *Egr1* displayed a substantial upregulation (Figures 7K, L). Since the switching of 3′ UTRs may have influenced protein expression rather than mRNA, the protein levels of Rala were further investigated through immunofluorescence analysis. Importantly, P7 mice exhibited markedly increased levels of *Rala* compared to P1 mice, while the alterations were reversed under the GF condition (Figure 8).

### Immune cell infiltration and correlation analysis

**FIGURE 9:**
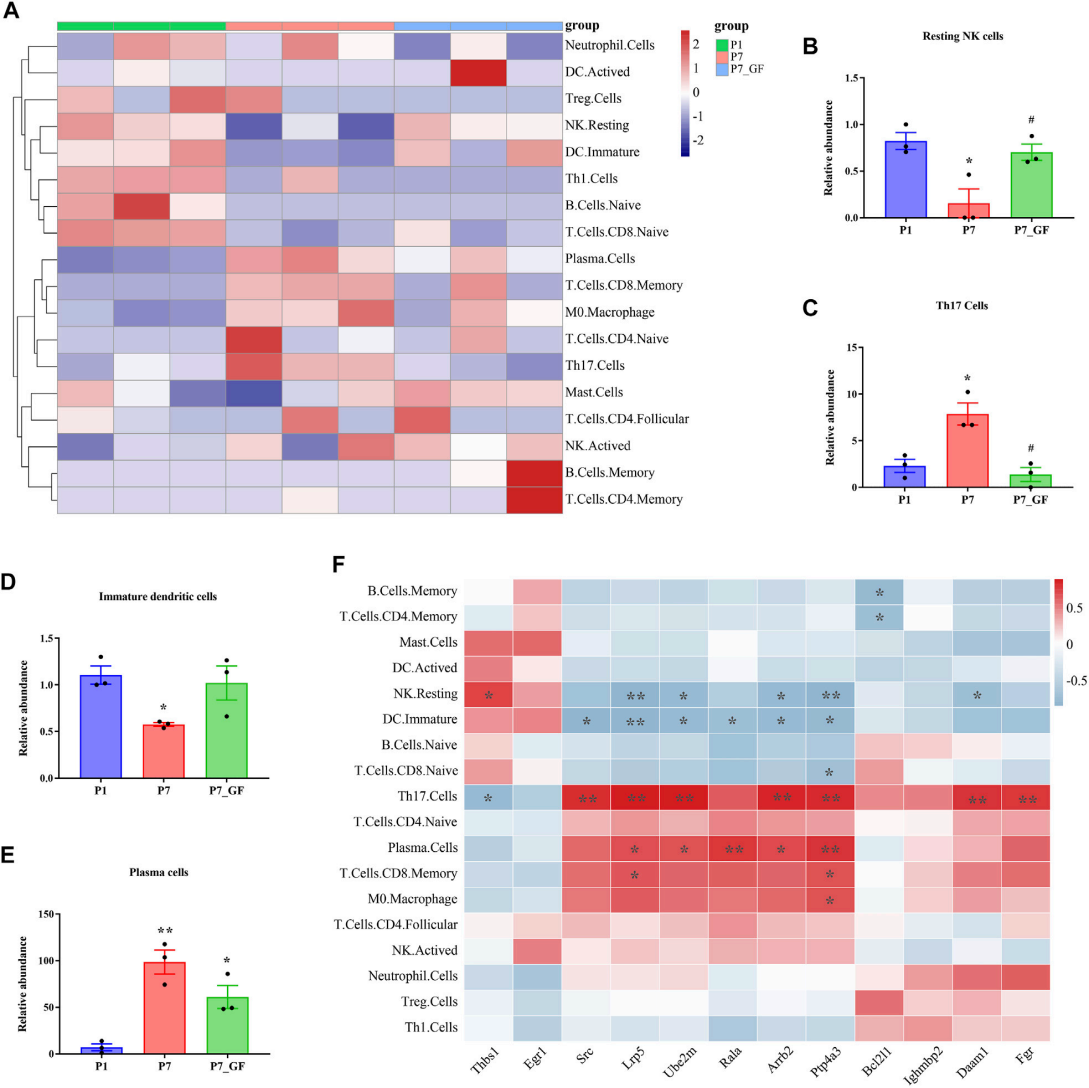
Immune cell infiltration and correlation analysis. **(A)** Overall, the three groups showed a difference in immune cells infiltrations, and the infiltrations in P7_GF mice appeared to be lower than those in the other two groups. Red represents high expression and blue low expression. **(B)** The resting NK cells were significantly decreased in P7 mice but completely restored in P7_GF mice. ^*^ *p* < 0.05 versus the P1 mice; ^#^ *p* < 0.05 versus the P7 mice. **(C)** The Th17 cells were notably increased in P7 mice but completely reversed in P7_GF mice. ^*^ *p* < 0.05 versus the P1 mice; ^#^ *p* < 0.05 versus the P7 mice. **(D)** The immature dendritic cells were significantly decreased in P7 mice. ^*^ *p* < 0.05 versus the P1 mice. **(E)** The plasma cells were significantly increased in P7 and P7_GF mice. ^*^ *p* < 0.05, ^**^ *p* < 0.01 versus the P1 mice. **(F)** These immune cells were significantly correlated to *Rala* and the related DEGs. ^*^ *p* < 0.05, ^**^ *p* < 0.01. Data were expressed as mean ± SEM (*n* = 3).

The composition of immune cell subtypes in the heart was assessed using the CIBERSORT algorithm. Overall, differences in immune cell infiltrations were observed among the three groups, with lower infiltrations evident in P7_GF mice as compared to the other two groups (Figure 9A). Notably, resting NK cells exhibited a decrease in P7 mice, but their levels were completely restored in P7_GF mice (Figure 9B). Conversely, Th17 cells were notably increased in P7 mice, but their levels were completely reversed in P7_GF mice (Figure 9C). Although immature dendritic cells and plasma cells displayed similar variations, the differences were not statistically significant (Figures 9D, E). Furthermore, these immune cell subsets showed significant correlations with *Rala* and the associated DEGs (Figure 9F). Resting NK cells were positively correlated with Thbs1 but exhibited significant negative correlations with *Lrp5, Ube2m, Arrb2, Ptp4a3,* and *Daam1*. In contrast, Th17 cells were negatively correlated with Thbs1 but showed prominent positive correlations with *Src*, *Lrp5*, *Ube2m*, *Arrb2*, *Ptp4a3*, *Daam1* and *Fgr*.

## Discussion

In this study, we present reliable evidence demonstrating a notable switch in tandem 3′ UTRs of cardiac genes at postnatal day 7 under GF conditions. Specifically, we observed significant alterations in the 3′ UTRs of mitochondria-related genes, namely *Rala, Timm13,* and *Uqcc3*. Furthermore, the GF condition resulted in a pronounced reduction in mitochondrial cristae density and decreased expression levels of Tomm20 in postnatal hearts. Additionally, the GF condition was associated with lower infiltration of immune cells, and the levels of resting NK cells, Th17 cells, immature dendritic cells, and plasma cells in P7_GF mice were found to be highly comparable to those in P1 mice. Taken together, our findings unequivocally indicate that the microbiota plays a crucial role in postnatal heart development by influencing APA switching, mitochondrial function, and immune cell infiltration.

Previous studies have demonstrated that the mammalian heart has the ability to fully regenerate damaged myocardium during early life; however, this regenerative capacity diminishes by postnatal day 7 (Porrello et al., 2011; Porrello et al., 2013; Puente et al., 2014; Notari et al., 2018) coinciding with the completion of heart development (Romanowicz et al., 2021). Consequently, our study focused on mice within the first 24 h after birth and at postnatal day 7. The loss of regenerative ability is believed to be a protective mechanism in response to the postnatal environment rich in oxygen (Puente et al., 2014). Notably, increases in extracellular matrix stiffness and expression of the miR-15 family have been implicated in this process (Porrello et al., 2013; Notari et al., 2018). Moreover, both microbiota (Wostmann et al., 1982; Guzzardi et al., 2019; Wang T. et al., 2021) and alternative splicing (Ladd et al., 2005; Xu et al., 2005; Giudice et al., 2014) have been shown to influence postnatal heart remodeling and development. In this study, we provide the first evidence of a close relationship between microbiota and APA during postnatal heart development. Through the temporal profiling of APA switching in mice with or without microbiota exposure, we uncovered the pivotal role of microbiota in regulating APA transitions and heart development. Strikingly, more than 70 percent of the APA switching genes in GF mice exhibited longer 3′ UTRs compared to their conventionally counterparts. Conversely, approximately two-thirds of the DEGs in GF mice were downregulated. It is worth noting that previous studies have indicated that lengthening of 3′ UTRs can result in reduced gene expression, while shortening of 3′ UTRs can stabilize mRNA and increase protein production (Mayr and Bartel, 2009; Chen et al., 2018). Consistent with these observations, our results demonstrated that the 3′ UTRs of *Rala* were lengthened, accompanied by decreased protein expression. Therefore, we hypothesize that the lengthening of tandem 3′ UTRs in response to the GF condition may contribute to a lower level of gene expression and protein production.

Furthermore, we identified that the genes with switched 3′ UTRs between P7 mice and their GF counterparts are significantly associated with myofibril assembly, mitochondrial membrane organization, and the Apelin signaling pathway. Previous studies have demonstrated that impaired myofibril assembly can adversely affect heart development (Fritz-Six et al., 2003) and is closely related to congenital heart defects (Granados-Riveron et al., 2010). Moreover, a deficiency in the Apelin signaling pathway has been shown to reduce the number of myocardial progenitor cells and impair heart development (Quertermous, 2007; Sagrac et al., 2020). Additionally, mitochondrial function has been recognized as crucial in myocyte differentiation and heart development (Lingan et al., 2017; Zhao et al., 2022). Notably, mitochondrial-dependent oxidative stress has been identified as a key mechanism for inducing cardiomyocyte cell-cycle arrest by causing DNA damage during the initial postnatal week (Puente et al., 2014). Considering the pivotal importance of mitochondria in postnatal heart development and maturation (Puente et al., 2014; Lingan et al., 2017; Zhao et al., 2022), we focused on genes enriched in the module associated with mitochondrial membrane organization. Among them, *Rala*, *Timm13*, and *Uqcc3* were identified as potential key regulatory genes, and their tandem 3′ UTRs were further confirmed. *Rala* has been found to be expressed in the embryonic myocardium (Zhao and Rivkees, 2000) and plays a crucial role in mitochondrial fission, segregation, and ATP generation (Kashatus et al., 2011; Chamberlain et al., 2022). Timm13 is essential for the import of proteins into the mitochondrial inner membrane by forming a complex with Timm8 in the intermembrane space (Paschen et al., 2000). Also, Uqcc3 has been identified to function as a component of mitochondrial complex III (Wanschers et al., 2014) and has been demonstrated to play a role in maintaining mitochondrial structure and function under hypoxic stress (Yang et al., 2020). Moreover, we observed a significant decrease in mitochondrial cristae density and a reduced level of Tomm20 in postnatal hearts under GF conditions. Tomm20 has been established as a marker for mitochondrial mass and oxidative phosphorylation (Curry et al., 2016). It is well-known that oxidative phosphorylation is a fundamental process that is crucial for cardiac maturation (Wildenthal, 1973). Therefore, our findings strongly suggest that the GF condition can impact mitochondrial structure and function during postnatal heart development, potentially through the disruption of APA switching mechanisms.

Additionally, the DEGs identified in our study contribute to enhancing our understanding of how the microbiota regulates postnatal heart development. Importantly, it is noteworthy that the DEGs between conventional mice and their GF counterparts exhibit substantial differences, thus leading to distinct functional features. Moreover, we discovered a close association between the DEGs and the validated APA switching genes. Specifically, *Rala* demonstrated a correlation with *Src* and further exhibited connections with other DEGs. *Src* is included in the gene set associated with the positive regulation of kinase activity, a process that ranks at the top of the list of biological processes enriched in the P7_vs_P7_GF group. Previous research has established the significant role of *Src* in promoting myocardial mitochondrial function, development, and regeneration (Peng et al., 2008; Feng et al., 2010; Missinato et al., 2015). In addition to *Src*, several other DEGs also have been involved in cardiomyocyte proliferation, heart function and development. For example, *Daam1* is necessary for the development of heart morphogenesis (Li et al., 2011), and *Arrb2* can promote ischemia-reperfusion injury and induce cardiomyocyte death (Wang et al., 2017). Similarly, *Lrp5* has been shown to play a crucial role in cardiomyocyte proliferation and neonatal heart regeneration (Zhou et al., 2022). Together, the disruptions of these DEGs in the postnatal heart may lead to major changes in the myocardial structure and function in GF mice.

Emerging evidence suggests that immune cells play a role in cardiac development and regeneration (Aurora et al., 2014; Sayers and Riley, 2021). Therefore, we further investigated the landscape of immune cell subtypes among the different groups. Overall, there appeared to be fewer infiltrating immune cells in GF mice. Notably, the levels of resting NK cells, Th17 cells, immature dendritic cells, and plasma cells in P7_GF mice were comparable to those in P1 mice, indicating that the GF condition may hinder immune system maturation during postnatal heart development. Resting NK cells can be rapidly activated by various cytokines, allowing them to acquire effector functions (Barber and Long, 2003). The increase of resting NK cells in P7_GF mice may be attributed to the absence of stimulation from the microbiota. In contrast, Th17 cells were significantly enriched in P7 mice but reduced in their GF counterparts. This finding aligns with a recent study demonstrating increased Th17 cells in 8-day-old mice. Moreover, it was shown that cytokines produced by Th17 cells can directly inhibit cell proliferation and promote apoptosis in neonatal cardiomyocytes (Li et al., 2020). Additionally, these immune cell populations exhibited significant correlations with the *Rala* and the related DEGs. This suggests that the APA switching genes and DEGs, differentially regulated by the microbiota, may impact immune system maturation and subsequently affect postnatal heart development. Nevertheless, we currently lack concrete evidence to exclude the possibility that immune cells are initially influenced by the microbiota, thereby disrupting APA switching during postnatal heart development. Therefore, further studies are warranted to determine the causal relationship between APA switching and immune cell infiltration in a GF condition.

## Conclusion

In summary, our findings provide reliable evidence highlighting the crucial role of microbiota in postnatal heart development through its impact on tandem 3′ UTR patterns, mitochondria, and immune cell infiltrations. To the best of our knowledge, this is the inaugural study investigating the influence of microbiota on heart development in juvenile mice using a comprehensive genome-wide analysis of APA site switching. We anticipate that this study will significantly enhance our understanding of the underlying mechanisms by which microbiota influences postnatal heart development and maturation.

## Data Availability

The datasets presented in this study can be found in online repositories. The names of the repository/repositories and accession number(s) can be found in the article/Supplementary Material.
