# Supplementary material for: Microbiota affects mitochondria and immune cell infiltrations via alternative polyadenylation during postnatal heart development
Source: Front Cell Dev Biol. 2024 Jan 12;11:1310409. doi: 10.3389/fcell.2023.1310409 (PMC10820713; doi:10.3389/fcell.2023.1310409)
Supplement: Supplementary file 1 [file DataSheet1.ZIP › Supplementary materials/Supplementary Table 1.docx]

**Supplementary Table 1**

qPCR primers (Mus musculus) used in the study.

| Name | Primer | Sequence (5’-3’) |
| --- | --- | --- |
| *Rala* (Proximal) | Forward Primer | CGAGCCAGAAAGATGGAAGACAG |
|  | Reserves Primer | GAGCCTCCAATGGCAATGCTTAT |
| *Rala* (Distal) | Forward Primer | CCACTTGCTGCCCATGTGACGAA |
|  | Reserves Primer | CTGTGCCCTGCTCCCAGGAGATT |
| *Vamp2* (Proximal) | Forward Primer | TCCCATCACCTCTCTCTCACC |
|  | Reserves Primer | GATCGGTTAAATCCAAGGGAAGG |
| *Vamp2* (Distal) | Forward Primer | ATGTGGGGCACTCAACTGAC |
|  | Reserves Primer | AGAACCAGATTGTGGTCGGC |
| *Timm13* (Proximal) | Forward Primer | TCTCCTCCTAGCCGCCCTGAAGT |
|  | Reserves Primer | ATCCCTGTGCTCTGTCGGGTCCTC |
| *Timm13* (Distal) | Forward Primer | CCAGCCGAGGTTAAGAAGCAAGG |
|  | Reserves Primer | AAGCCACGAGTCTCCAGGAGCAG |
| *Uqcc3* (Proximal) | Forward Primer | TTTTCGCCCAGAATGCAAACATC |
|  | Reserves Primer | CGTCCTTCAGGGTAGCCATCACC |
| *Uqcc3* (Distal) | Forward Primer | ATGGAGTCGGGAGATACACCAGG |
|  | Reserves Primer | TTTTACCCAACAGTTGCAGAAGCA |
| *Gapdh* | Forward Primer | ACTCTTCCACCTTCGATGCC |
|  | Reserves Primer | TGGGATAGGGCCTCTCTTGC |

qPCR, quantitative polymerase chain reaction; *Rala*, v-ral simian leukemia viral oncogene A (ras related); *Vamp2*, vesicle-associated membrane protein 2; *Timm13*, translocase of inner mitochondrial membrane 13; *Uqcc3*, ubiquinol-cytochrome c reductase complex assembly factor 3; *Gapdh*, glyceraldehyde-3-phosphate dehydrogenase.
