# Supplementary material for: Microbiota affects mitochondria and immune cell infiltrations via alternative polyadenylation during postnatal heart development
Source: Front Cell Dev Biol. 2024 Jan 12;11:1310409. doi: 10.3389/fcell.2023.1310409 (PMC10820713; doi:10.3389/fcell.2023.1310409)
Supplement: Supplementary file 1 [file DataSheet1.ZIP › Supplementary materials/Supplementary Table 2.docx]

**Supplementary Table 2**

Summary of the alternative polyadenylation sequencing data.

|  | **P1_1** | **P1_2** | **P1_3** | **P7_1** | **P7_2** | **P7_3** | **P7_GF_1** | **P7_GF_2** | **P7_GF_3** | **Combined (%)** |
| --- | --- | --- | --- | --- | --- | --- | --- | --- | --- | --- |
| **Raw reads** | 12927608 | 16074331 | 14762275 | 16417092 | 17485677 | 35585270 | 20293507 | 30798967 | 17420798 | 181765525 (100.0) |
| **Qualified reads** | 12918067 | 16064964 | 14753938 | 16389750 | 17449793 | 35506968 | 20278223 | 30781694 | 17404531 | 181547928 (99.9) |
| **Mapped to genome** | 12130235 | 14826134 | 13778123 | 15097109 | 16007140 | 32705671 | 18779030 | 28362291 | 16163067 | 167848800 (92.3) |
| **Uniquely mapped to genome** | 8579453 | 10478903 | 9723879 | 10537054 | 11212228 | 22698922 | 13047534 | 19641245 | 11147557 | 117066775 (64.4) |
| **Mapped to nuclear genome** | 6846592 | 8338536 | 7576320 | 8014846 | 8668861 | 17430784 | 9762396 | 15162285 | 8417780 | 90218400 (49.6) |
| **After internal priming filter** | 5393040 | 6574257 | 5972034 | 6507116 | 7049076 | 14145590 | 7879860 | 12045758 | 6695051 | 72261782 (39.8) |
| **Genes sampled by reads** | 16047 | 16300 | 16018 | 16057 | 16194 | 17018 | 16115 | 16791 | 15980 | 146520 (0.1) |
| **Cleavage clusters** | 156320 | 170675 | 161395 | 147077 | 154050 | 232686 | 167276 | 230753 | 170547 | 1590779 (0.9) |
| **Known polyA sites sampled** | 18813 | 19587 | 19102 | 19462 | 19703 | 21742 | 19952 | 21338 | 19494 | 179193 (0.1) |
| **Putative novel polyA sites** | 137507 | 151088 | 142293 | 127615 | 134347 | 210944 | 147324 | 209415 | 151053 | 1411586 (0.8) |
| **Genes sampled by cleavage clusters** | 14439 | 14782 | 14463 | 14368 | 14517 | 15430 | 14780 | 15403 | 14561 | 132743 (0.1) |
