# Supplementary figures and images for: Microbiota affects mitochondria and immune cell infiltrations via alternative polyadenylation during postnatal heart development
Source: Front Cell Dev Biol. 2024 Jan 12;11:1310409. doi: 10.3389/fcell.2023.1310409 (PMC10820713; doi:10.3389/fcell.2023.1310409)

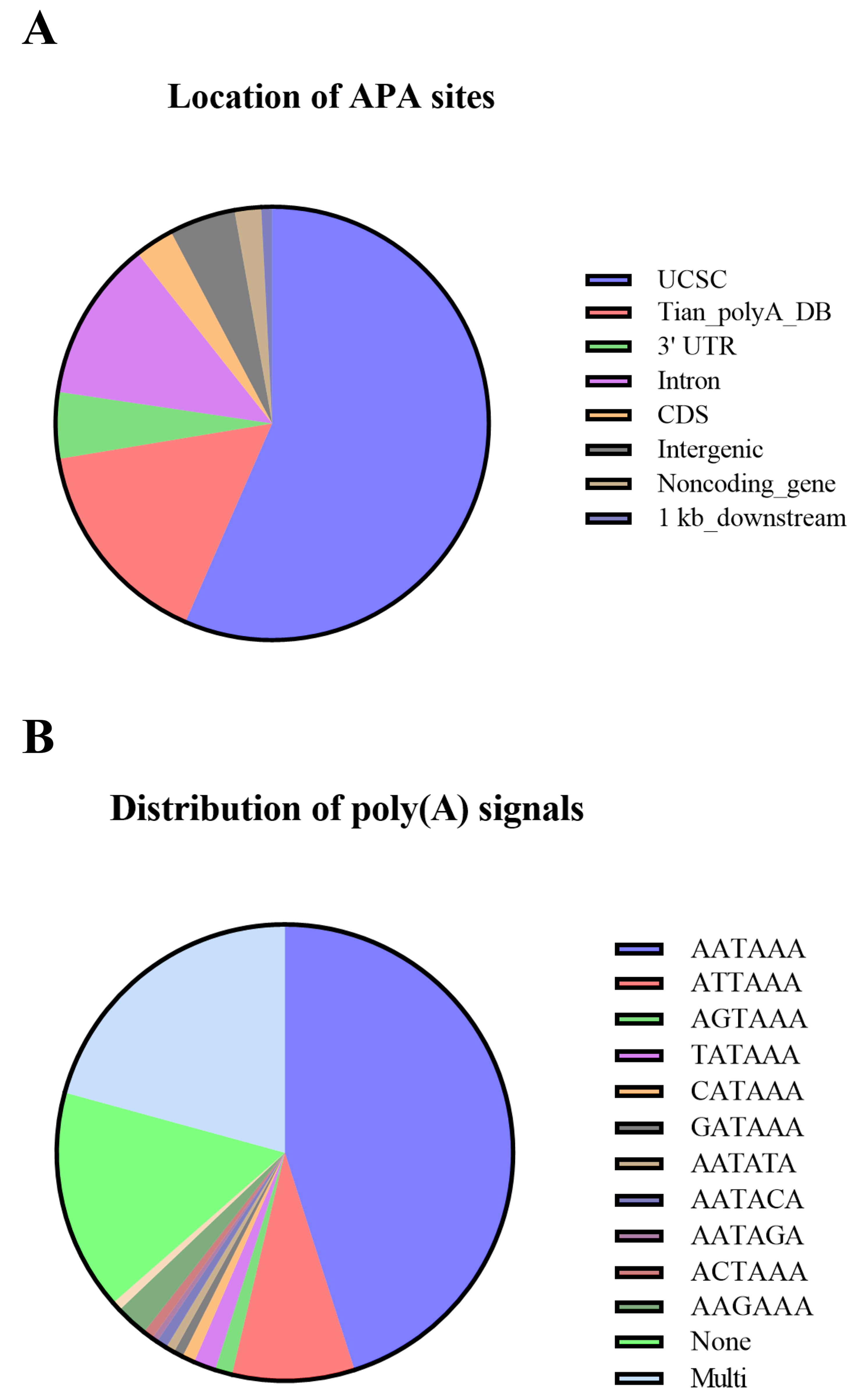

Supplement: Supplementary file 1 [file DataSheet1.ZIP › Supplementary materials/Supplementary Figure 1.jpg]

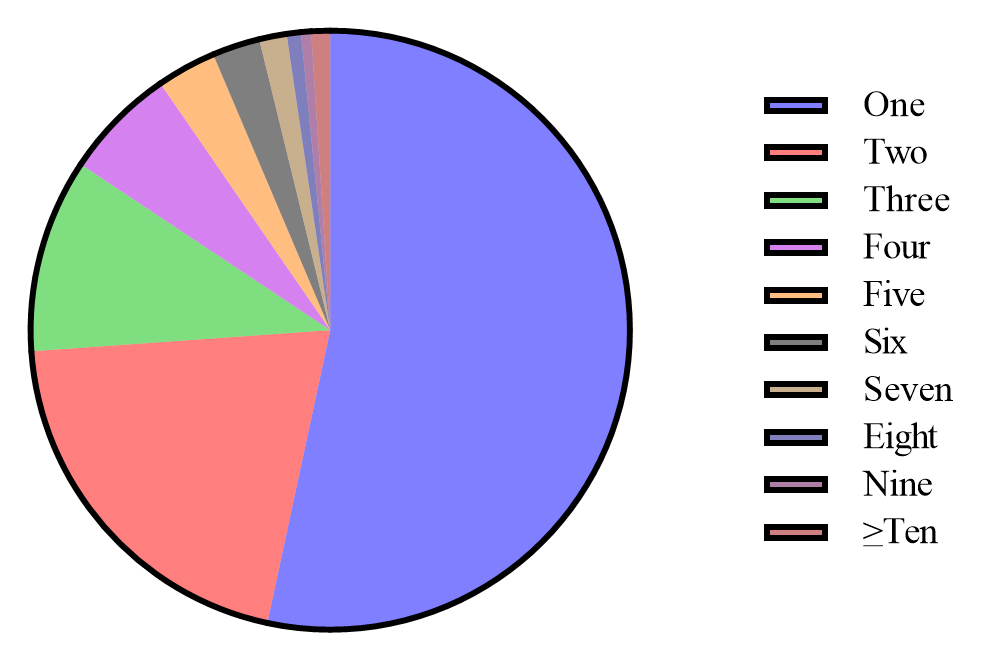

Supplement: Supplementary file 1 [file DataSheet1.ZIP › Supplementary materials/Supplementary Figure 2.tif]
